# Supplementary material for: Symmetry breaking of tissue mechanics in wound induced hair follicle regeneration of laboratory and spiny mice
Source: Nat Commun. 2021 May 10;12:2595. doi: 10.1038/s41467-021-22822-9 (PMC8110808; doi:10.1038/s41467-021-22822-9)
Supplement: Supplementary file 1 — Supplementary Information [file 41467_2021_22822_MOESM1_ESM.pdf]

Supplementary Information

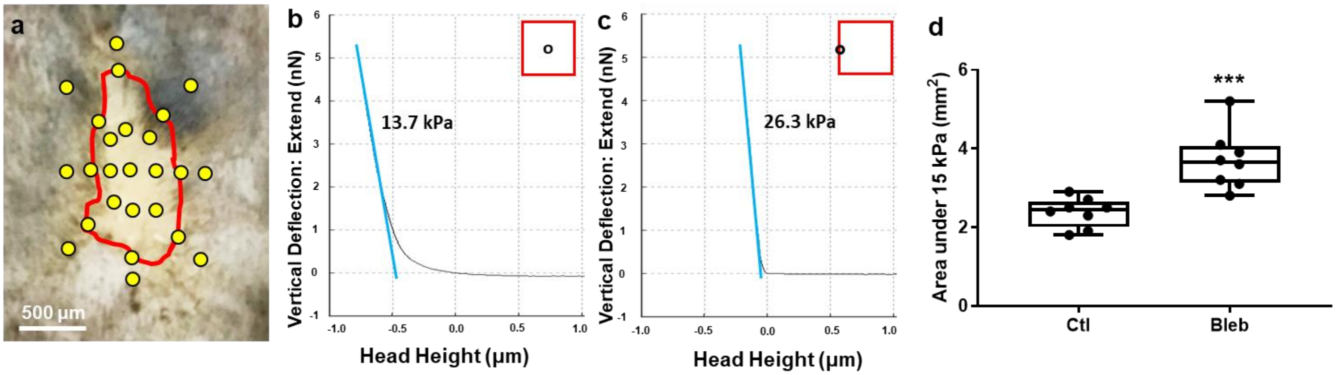

Supplementary Fig 1. Measuring stiffness of the wound using AFM. a, Illustration of AFM measurements on the respective location of the wound. Yellow dots indicate the location of measurement. Red line demarcates the boarder of the wound. **b-c**, Representative force-distance curve obtained from measurement at the wound center or margin. Red box: wound; black dot: location of the measurement on the wound. Blue line: linear region used to calculate the slope of the force curve. **d**, Graph showing the area of the wound stiffness under 15 kPa in control and Blebbistatin treated wounds at PWD14. In the box plot, center lines show the medians; box limits indicate the 25th and 75th percentiles; whiskers extend 1.5 times the interquartile range. $p = 0.0005$, unpaired two-sided *t*-test. $n = 8$ independent biological animals. The image **a** represents 8 out of 8 experiments.

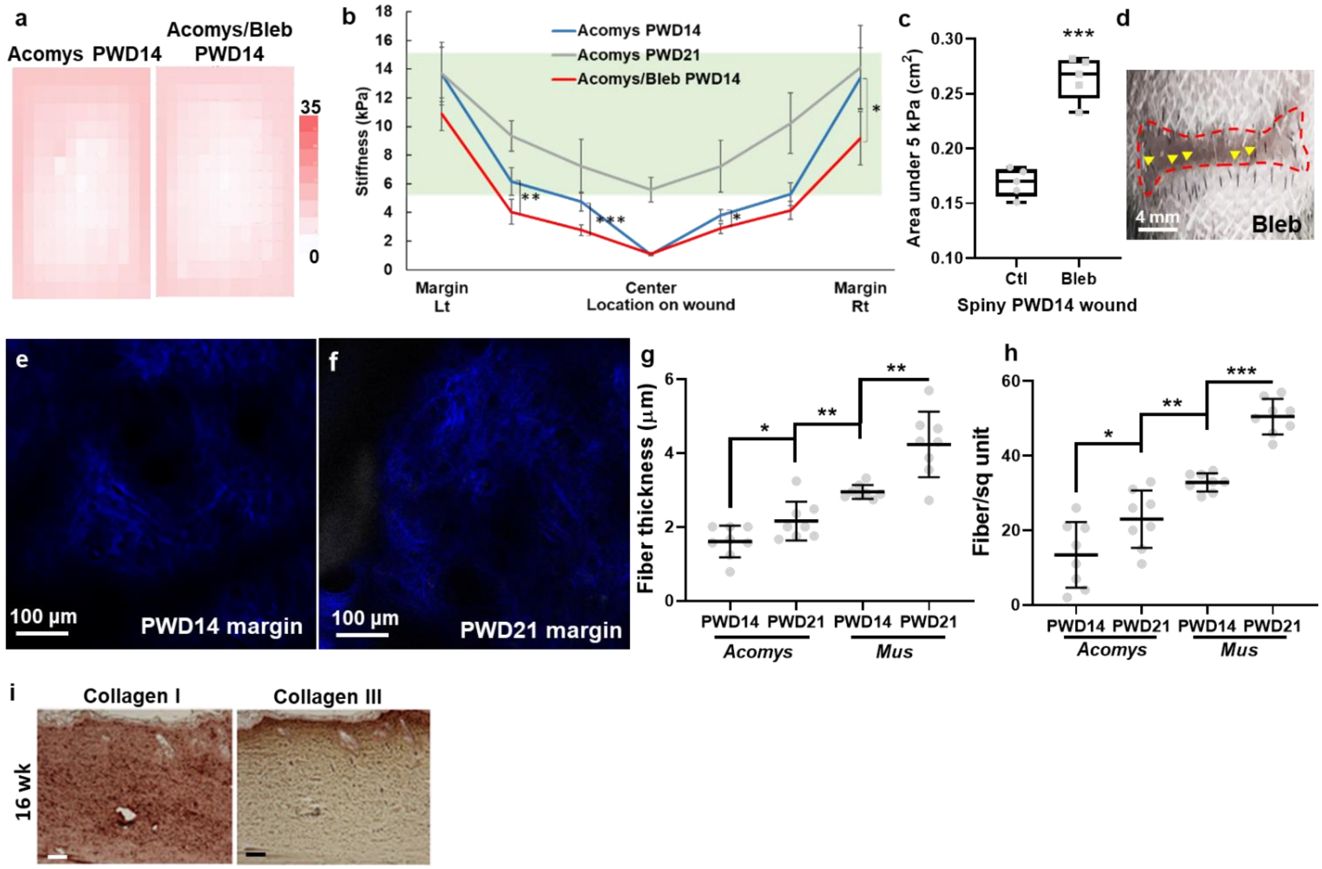

Supplementary Fig 2. The mechanical characterization of the spiny mouse wound. a, Stiffness heatmap of control and Blebbistatin treated wounds. Colorimetric scale unit: kPa. **b**, Spatial stiffness graph of PWD14, PWD21 and PWD14-Blebbistatin treated Acomys wounds. Green box: morphogenetic range. n = 10 regions examined over 5 biologically independent animals per location per condition. Data are presented as mean values ± SD. Comparison between Acomys PWD14 and Acomys/Bleb PWD14, p = 0.0381 at margin right, p = 0.0421 at center-right; **, p = 0.0089; ***, p < 0.0001. One-way ANOVA, Tukey test. **c**, Area under 5 kPa in control and Blebbistatin-treated PWD14 Acomys wounds. In the box plot, center lines show the medians; box limits indicate the 25th and 75th percentiles; whiskers extend 1.5 times the interquartile range. p < 0.0001, unpaired two-sided *t*-test. n = 5 independent biological animals. **d**, Representative photo of Blebbistatin-treated Acomys wound on PWD28. Yellow arrows indicate the newly formed hair fibers. Red broken line demarcates the wound margin. **e-f**, Collagen fibrils imaged by SHG at the wound margin of PWD14 and PWD28 Acomys wounds, respectively. **g-h**, Dot plots of fiber thickness and fiber density, respectively, quantified from SHG images taken from wound margin of the spiny and laboratory mice on PWD14 and PWD28. Fiber thickness: comparison between Acomys PWD14-PWD21: p = 0.0368; between Acomys PWD21-Mus PWD14: p = 0.0013; between Mus PWD14-PWD21: p = 0.0013. Fiber/sq unit: comparison between Acomys PWD14-PWD21: p = 0.0361; between Acomys PWD21-Mus PWD14: p = 0.0037; between Mus PWD14-PWD21: p < 0.0001. Data are presented as mean values ± SD. n = 8 regions examined over 4 biologically independent animals. One-way ANOVA, Tukey test. **i**, IHC of collagen I and III in 16-week post-operation spiny mouse wounds. The image represents 5 out of 5 experiments performed. Scale: 100 µm. The image **a**, **d-f** represent 4 out of 4 experiments.

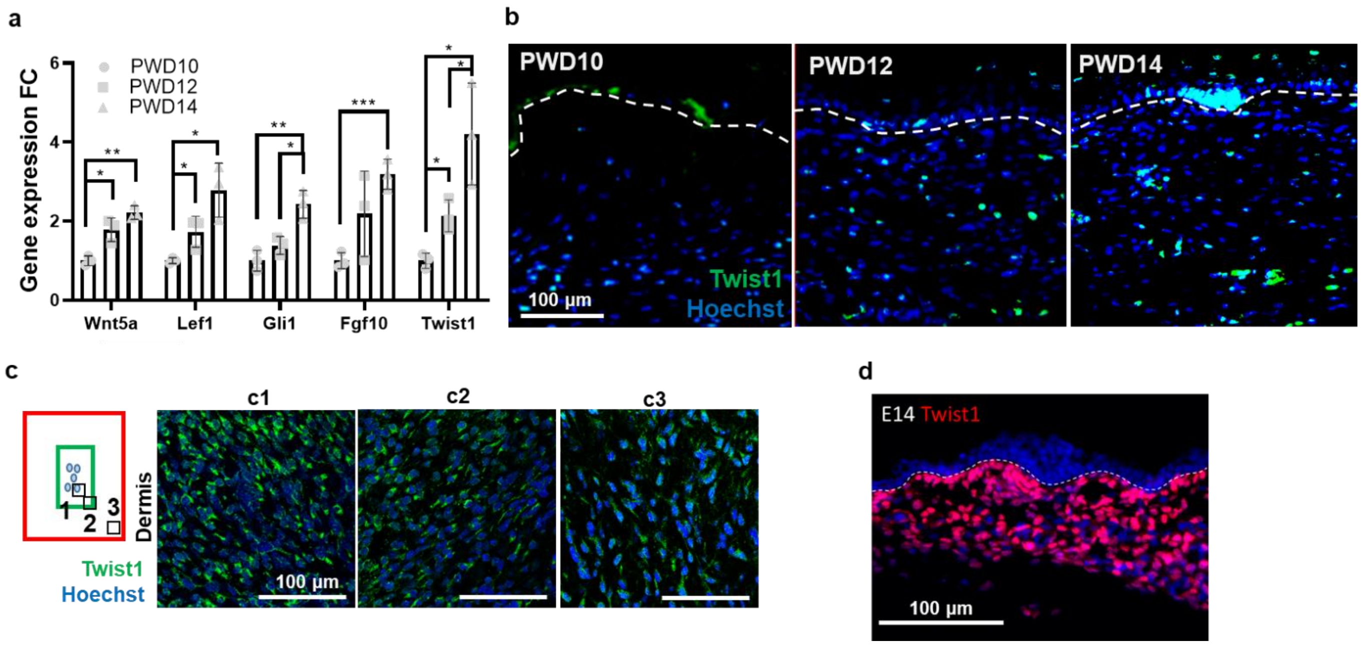

Supplementary Fig 3. **Expression of Twist1 related molecules during WIHN and skin development. a**, qPCR gene expression FC of epidermis wound center harvested from PWD10, 12, and 14 laboratory mice. Wnt5a: comparison between PWD10-PWD12, p = 0.014; between PWD10-PWD14, p = 0.0005. Lef1: comparison between PWD10-PWD12, p = 0.0335; between PWD10-PWD14, p = 0.0107. Gli1: comparison between PWD10-PWD14, p = 0.0048; between PWD12-PWD14, p = 0.0124. Fgf10: comparison between PWD10-PWD14, p = 0.0009. Twis1: comparison between PWD10-PWD12, p = 0.0119; between PWD10-PWD14, p = 0.013; between PWD12-PWD14, p = 0.0133. n = 3 biologically independent experiments performed. Data are presented as mean values ± SEM. One-way ANOVA, Tukey test. **b**, IHC of Twist1 of PWD10, 12 and 14 wounds of laboratory mice. **c**, Wholemount immunostaining of dermal Twist1 of PWD14 from wound center **c1**, between wound center and margin **c2** and wound margin **c3** regions in the dermis of laboratory mice. The illustration shows the respective location imaged from the wound. Red line: wound margin. Green: morphogenetic zone. Blue dots: de novo hair follicles. **d**, IHC of E14 embryonic laboratory mouse dorsal skin stained against Twist1 and Hoechst. Dotted line demarcates the epidermal-dermal junction. Blue: nucleus. The image **b-d** represent 4 out of 4 experiments.

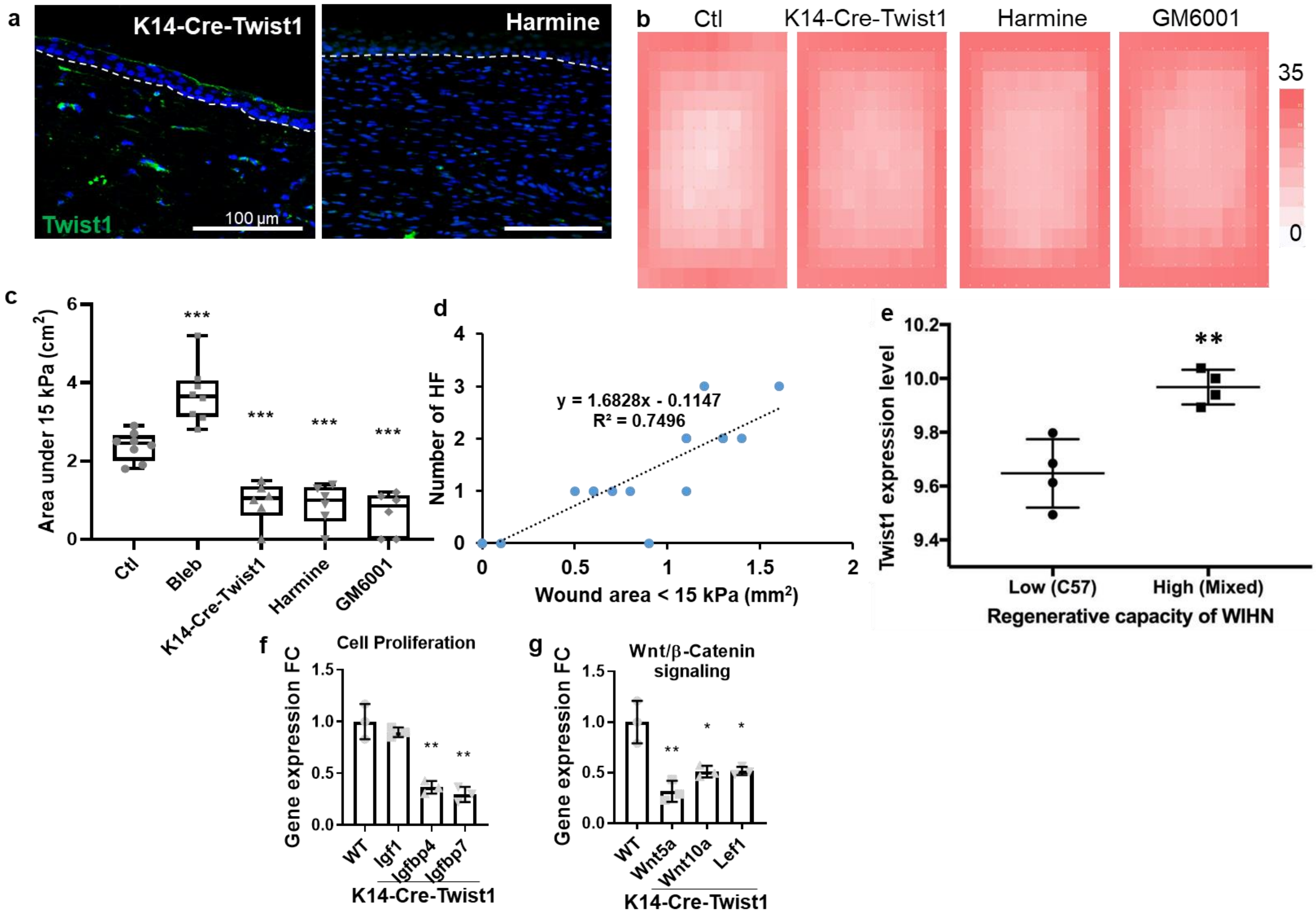

Supplementary Fig 4. The effects of Twist1-perturbation on wound stiffness and gene expression. a, IHC of PWD14 K14-Cre-Twist1 and Harmine-treated wounds stained against Twist1 and Hoechst. **b**, Stiffness heatmaps of the laboratory mouse wounds under different perturbations. Scale bar unit: kPa. **c**, Changes in wound bed area of stiffness under 15 kPa in various conditions. In the box plot, center lines show the medians; box limits indicate the 25th and 75th percentiles; whiskers extend 1.5 times the interquartile range. All comparisons were made to Ctl. Bleb: p = 0.0005; K14-Cre-Twist1, Harmine and GM6001: p < 0.0001. Unpaired two-sided *t*-test. n = 6 biologically independent animals. **d**, Correlation graph of the wound area under 15 kPa and the resultant number of hair follicles. **e**, Twist1 is expressed higher in the wounds of the high regenerative capacity mouse strain. n = 4 biologically independent animals. P = 0.004, Unpaired two-sided *t*-test. Data are presented as mean values ± SEM. Mixed: C57BL/6 X FVB X SJL mice. Microarray data from Nelson et al[1]. **f** and **g**, qPCR analysis showing gene expression fold change (FC) related to cell proliferation and Wnt/β-catenin signaling, respectively, in PWD14 epidermis wound center in wild type vs K14-Cre-Twist1 comparison. n = 3 biologically independent samples. Data are presented as mean values ± SEM. All comparisons made to WT. *Igfbp4*, p = 0.0037; *Igfbp7*, p = 0.0028. *Mmp13*, p = 0.0005; *Itga1*, p = 0.0071. *Wnt5a*, p = 0.0073; *Wnt10a*, p = 0.0177; *Lef1*, p = 0.0175. Unpaired two-sided *t*-test. The images **a** represent 4 out of 4 experiments. The images **b** represent 6 out of 6 experiments.

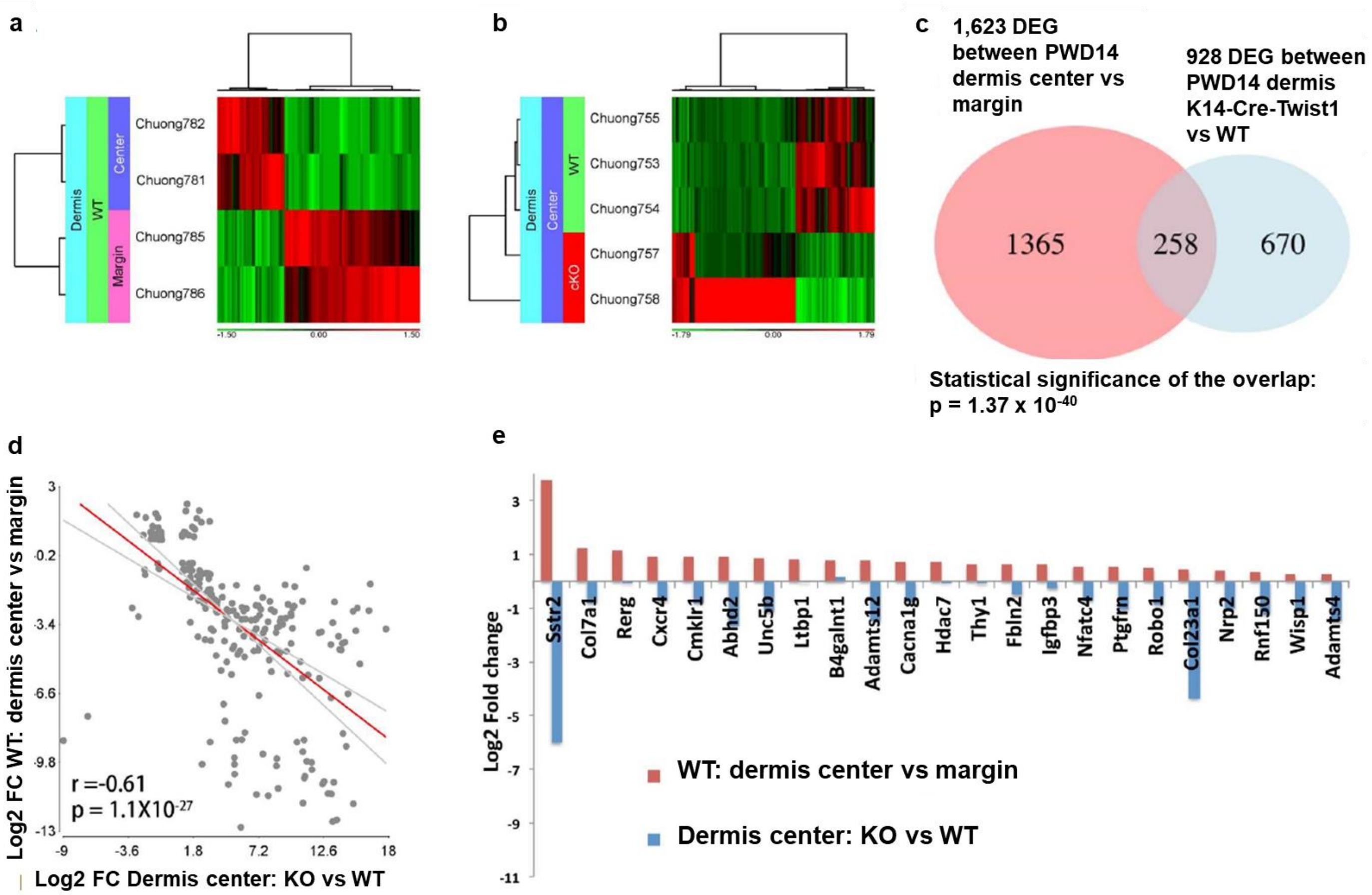

Supplementary Fig 5. Identifying epidermal Twist1 as a key regulatory gene during WIHN and dermal condensation in both epidermis and dermis. a, Hierarchical clustering of 1623 differentially expressed genes between wild type dermal wound center and wild type dermis wound margin. n = biologically independent experiments. **b**, Hierarchical clustering of 928 differentially expressed genes between K14-Cre-Twist1 dermis wound center and wild type dermis wound center. n = 2 and 3 biologically independent experiments in knockout and wild type mice, respectively. **c**, Venn diagram showing overlapping DEGs from **a** and **b** ($p = 1.37 \times 10^{-40}$). **d**, The gene expression levels of WIHN associated genes that are upregulated in dermis wound center (compared to dermis wound margin) are decreased by the loss of Twist1 in epidermis, and vice versa. The 258 gray dots represent 258 overlapping DEG from **c**. The red and gray lines indicate the regression line and 95% confidence interval lines, respectively. Pearson correlation = -0.61, and one-tailed $p = 1.1 \times 10^{-27}$. **e**, Dermal condensation (DC) signature genes were upregulated in wild-type dermis wound center (compared to margin), which were downregulated in K14-Cre-Twist1.

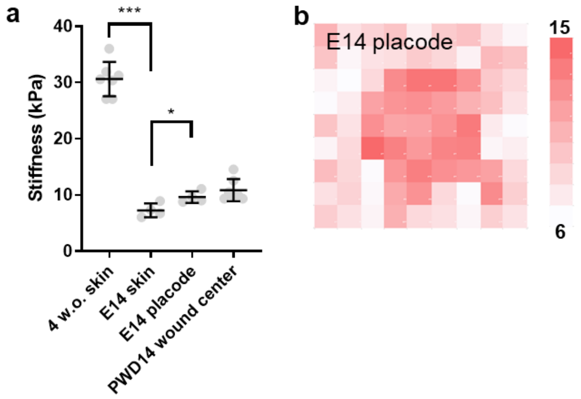

Supplementary Fig 6. Relative stiffness of adult skin, embryonic skin, developing hair placode and PWD14 wound center. a, Stiffness of 4-week-old (w.o.) skin, E14 skin, E14 placode, and PWD14 wound center. Data are presented as mean values ± SEM. *, p = 0.0264. ***, p < 0.0001. Unpaired two-sided *t*-test. n = 4 biologically independent animals in E14 skin and E14 placode. n = 7 biologically independent animals in 4 w.o. skin and PWD14 wound center. **b**, Stiffness heatmap of E14 hair placode. Colorimetric unit: kPa. The image B represents 3 out of 3 experiments.

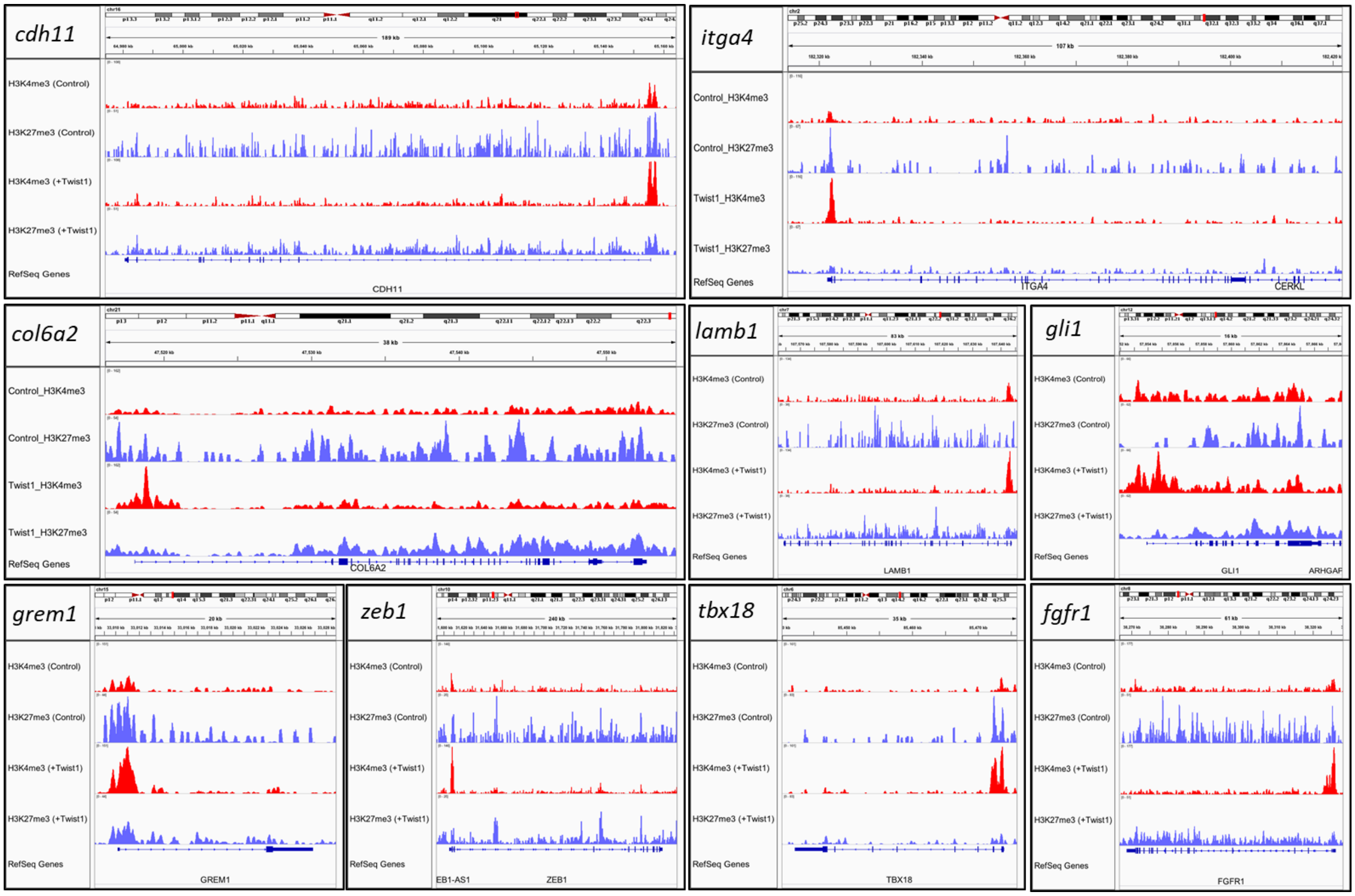

Supplementary Fig 7. H3K4me3 ChIP-seq analysis showing epigenetic reprogramming following Twist1-mediated overexpression in human epithelial cells. Twist1 is shown to directly regulate *Cdh11*, *Grem1*, *Zeb1*, *Dkk3*, *Gli1*, *Fgfr1*, *Tbox18*, *Col6a2*, and *Lamb1*. Figures are generated by re-analyzing database from Malouf et al[2].

Supplementary Table 1. List of bulk-RNA-seq samples

Sample	Species	PWD	Tissue	Region	Treat	RNA-seq read type
Chuong806	Lab mouse	14	Epidermis	Center	WT	Strand-specific paired-end 75bp
Chuong808	Lab mouse	14	Epidermis	Center	WT	Strand-specific paired-end 75bp
Chuong812	Lab mouse	14	Epidermis	Margin	WT	Strand-specific paired-end 75bp
Chuong813	Lab mouse	14	Epidermis	Margin	WT	Strand-specific paired-end 75bp
Chuong781	Lab mouse	14	Dermis	Center	WT	Strand-specific single-end 75bp
Chuong782	Lab mouse	14	Dermis	Center	WT	Strand-specific single-end 75bp
Chuong785	Lab mouse	14	Dermis	Margin	WT	Strand-specific single-end 75bp
Chuong786	Lab mouse	14	Dermis	Margin	WT	Strand-specific single-end 75bp
Chuong753	Lab mouse	14	Dermis	Center	WT	Strand-specific single-end 75bp
Chuong754	Lab mouse	14	Dermis	Center	WT	Strand-specific single-end 75bp
Chuong755	Lab mouse	14	Dermis	Center	WT	Strand-specific single-end 75bp
Chuong757	Lab mouse	14	Dermis	Center	KO	Strand-specific single-end 75bp
Chuong758	Lab mouse	14	Dermis	Center	KO	Strand-specific single-end 75bp
Chuong536	Spiny mouse	0	Epidermis	N.A.	WT	Non strand-specific single-end 75bp
Chuong541	Spiny mouse	15	Epidermis	N.A.	WT	Non strand-specific single-end 75bp
Chuong542	Spiny mouse	15	Epidermis	N.A.	WT	Non strand-specific single-end 75bp
Chuong545	Spiny mouse	20	Epidermis	N.A.	WT	Non strand-specific single-end 75bp
Chuong546	Spiny mouse	20	Epidermis	N.A.	WT	Non strand-specific single-end 75bp
Chuong538	Spiny mouse	30	Epidermis	N.A.	WT	Non strand-specific single-end 75bp
Chuong533	Spiny mouse	0	Dermis	N.A.	WT	Non strand-specific single-end 75bp
Chuong534	Spiny mouse	0	Dermis	N.A.	WT	Non strand-specific single-end 75bp
Chuong543	Spiny mouse	15	Dermis	N.A.	WT	Non strand-specific single-end 75bp
Chuong547	Spiny mouse	20	Dermis	N.A.	WT	Non strand-specific single-end 75bp
Chuong548	Spiny mouse	20	Dermis	N.A.	WT	Non strand-specific single-end 75bp
Chuong537	Spiny mouse	30	Dermis	N.A.	WT	Non strand-specific single-end 75bp

PWD: post-wound day; WT: wild type; KO: K14-Cre-Twist1 conditional knockout; N.A.: not available.

Supplementary Table 2. Primer table

Gene	Forward Primer	Reverse Primer
Cdh1	CAGTTCCGAGGTCTACACCTT	CAGTTCCGAGGTCTACACCTT
Cdh3	CCATTAGCGTCATATCCAGTGG	CATCAAGGATTTGCACAACGG
Col1a1	GCTCCTCTTAGGGGCCACT	ATTGGGGACCCTTAGGCCAT
Col4a1	CTGGCACAAAAGGGACGAG	ACGTGGCCGAGAATTTCACC
HGRT	TCAGTCAACGGGGGACATAAA	TCAGTCAACGGGGGACATAAA
Igf1	CACATCATGTCGTCTTCACACC	GGAAGCAACACTCATCCACAATG
Igfbp4	AGAAGCCCCTGCGTACATTG	TTGTTGGGATGTTCGCTCTCA
Igfbp7	AAGAGGCGGAAGGGTAAAGC	TGGGGTAGGTGATGCCGTT
Itga1	TCAAGTTGTGAACTCGTTCGC	GGGCCAATGTCCATCCTCTTA
Lef1	AACGAGTCCGAAATCATCCCA	AACGAGTCCGAAATCATCCCA
Mmp2	CATCAAGGATTTGCACAACGG	CTTCCGCATGGTCTCGATG
Mmp9	GCAGAGGCATACTTGTACCG	GCAGAGGCATACTTGTACCG
Mmp13	TGTTTGCAGAGCACTACTTGAA	TGTTTGCAGAGCACTACTTGAA
Snai1	CACACGCTGCCTTGTGTCT	GGTCAGCAAAAGCACGGTT
Snai2	CAGCGAACTGGACACACACA	ATAGGGCTGTATGCTCCCGAG
Twist1	ATAGGGCTGTATGCTCCCGAG	CGGAGAAGGCGTAGCTGAG
Wnt5a	ATGCAGTACATTGGAGAAGGTG	CGTCTCTCGGCTGCCTATTT
Wnt10a	CATGCTCGAATGAGACTCCAC	CATGCTCGAATGAGACTCCAC
Zeb1	AACGAGTCCGAAATCATCCCA	GGCGAGGAACACTGAGATGT
Zeb2	CCACGCAGTGAGCATCGAA	CAGGTGGCAGGTCATTTTCTT

Supplementary references

1 Nelson, A. M. *et al.* dsRNA Released by Tissue Damage Activates TLR3 to Drive Skin Regeneration. *Cell Stem Cell* **17**, 139-151, doi:10.1016/j.stem.2015.07.008 (2015).

2 Malouf, G. G. *et al.* Architecture of epigenetic reprogramming following Twist1-mediated epithelial-mesenchymal transition. *Genome Biol* **14**, R144, doi:10.1186/gb-2013-14-12-r144 (2013).
