# Supplementary material for: Symmetry breaking of tissue mechanics in wound induced hair follicle regeneration of laboratory and spiny mice
Source: Nat Commun. 2021 May 10;12:2595. doi: 10.1038/s41467-021-22822-9 (PMC8110808; doi:10.1038/s41467-021-22822-9)
Supplement: Supplementary file 2 — Reporting Summary [file 41467_2021_22822_MOESM2_ESM.pdf]

nature research

Corresponding author(s): Hans I-Chen Harn
Cheng-Ming Chuong

Last updated by author(s): Mar 3, 2020

Reporting Summary

Nature Research wishes to improve the reproducibility of the work that we publish. This form provides structure for consistency and transparency in reporting. For further information on Nature Research policies, see our Editorial Policies and the Editorial Policy Checklist.

Statistics

For all statistical analyses, confirm that the following items are present in the figure legend, table legend, main text, or Methods section.

n/a	Confirmed	
☐	☒	The exact sample size (n) for each experimental group/condition, given as a discrete number and unit of measurement
☐	☒	A statement on whether measurements were taken from distinct samples or whether the same sample was measured repeatedly
☐	☒	The statistical test(s) used AND whether they are one- or two-sided<br>*Only common tests should be described solely by name; describe more complex techniques in the Methods section.*
☐	☒	A description of all covariates tested
☐	☒	A description of any assumptions or corrections, such as tests of normality and adjustment for multiple comparisons
☐	☒	A full description of the statistical parameters including central tendency (e.g. means) or other basic estimates (e.g. regression coefficient) AND variation (e.g. standard deviation) or associated estimates of uncertainty (e.g. confidence intervals)
☐	☒	For null hypothesis testing, the test statistic (e.g. F, t, r) with confidence intervals, effect sizes, degrees of freedom and P value noted<br>*Give P values as exact values whenever suitable.*
☐	☒	For Bayesian analysis, information on the choice of priors and Markov chain Monte Carlo settings
☐	☒	For hierarchical and complex designs, identification of the appropriate level for tests and full reporting of outcomes
☐	☒	Estimates of effect sizes (e.g. Cohen's d, Pearson's r), indicating how they were calculated

Our web collection on statistics for biologists contains articles on many of the points above.

Software and code

Policy information about availability of computer code

Data collection	No software was used to collect data.
Data analysis	RNA-seq analysis: Partek Genomics Suite 7.18.0723; STAR 2.6.1d; htseq-count 0.6.0; TMM; edgeR 3.26.8; mouse mm10 reference genome, and RefSeq genome annotation (UCSC Genome Browser, 5 June, 2019); QIAGEN IPA (Content Version: 60467501, Build: ing_beryl, Date: 11-20-2020). Tissue stiffness: JPK package software (Data Processing, 6.3.11); MATLAB (R2015b). Wound area: ImageJ/Fiji (1.46). qPCR: pyQPCR (0.9).

For manuscripts utilizing custom algorithms or software that are central to the research but not yet described in published literature, software must be made available to editors and reviewers. We strongly encourage code deposition in a community repository (e.g. GitHub). See the Nature Research guidelines for submitting code & software for further information.

Data

Policy information about availability of data

All manuscripts must include a data availability statement. This statement should provide the following information, where applicable:

- Accession codes, unique identifiers, or web links for publicly available datasets
- A list of figures that have associated raw data
- A description of any restrictions on data availability

Bulk RNA-seq data are accessible at NCBI Gene Expression Omnibus (GEO) database (accession number: GSE159939) and can be downloaded from https://www.ncbi.nlm.nih.gov/geo/query/acc.cgi?acc=GSE159939

Field-specific reporting

Please select the one below that is the best fit for your research. If you are not sure, read the appropriate sections before making your selection.

☒ Life sciences ☐ Behavioural & social sciences ☐ Ecological, evolutionary & environmental sciences

For a reference copy of the document with all sections, see nature.com/documents/nr-reporting-summary-flat.pdf

Life sciences study design

All studies must disclose on these points even when the disclosure is negative.

Sample size	The Kolmogorov–Smirnov tests were conducted to test normal distributed random samples. We have calculated sample size based on a 95% confidence level to determine the minimal number of sample size necessary to obtain sufficient statistical power. The number is determined to be three replicates for RT-PCR, two replicates for bulk RNA-seq, and three replicates for control and knock-out experiments. Hence, 3 rounds of 2 or 3 mice per control/experimental group were carried out to achieve the final sample size of 6-8 in this study. Independent triplicates were used for RT-PCR. Independent duplicates were used for bulk-RNA-seq.
Data exclusions	No data was excluded
Replication	3 independent rounds of 2 or 3 mice per control/experimental group were carried out to achieve the final sample size of 6-8 in this study. Independent triplicates were used for RT-PCR. Independent duplicates were used for bulk-RNA-seq.
Randomization	Animals were randomly selected
Blinding	Blinding was applied during data quantification and interpretation of morphological changes of the wound and histology sections.

Reporting for specific materials, systems and methods

We require information from authors about some types of materials, experimental systems and methods used in many studies. Here, indicate whether each material, system or method listed is relevant to your study. If you are not sure if a list item applies to your research, read the appropriate section before selecting a response.

Materials & experimental systems

n/a	Involved in the study	
☐	☒	Antibodies
☐	☒	Eukaryotic cell lines
☒	☐	Palaeontology and archaeology
☐	☒	Animals and other organisms
☒	☐	Human research participants
☒	☐	Clinical data
☒	☐	Dual use research of concern

Methods

n/a	Involved in the study	
☒	☐	ChIP-seq
☒	☐	Flow cytometry
☒	☐	MRI-based neuroimaging

Antibodies

Antibodies used	Twist1 (ab50887, Cambridge, 1:50), Collagen I (ab34710, Cambridge, 1:50), Collagen III (ab7778, Cambridge, 1:50), MMP9 (N2C1, GTX100458, GeneTex, 1:50), Snai1 (13099-1-AP, Proteintech, 1:50), P-cadherin (13773-1-AP, Proteintech, 1:50), E-cadherin (20874-1-AP, Proteintech, 1:50) and Zeb2 (14026-1-AP, Proteintech, 1:50).

Validation	Twist1 (ab50887): validated to react with mouse by the manufacturer; applied in mouse IHC-P by Bai et al., Development 140:3395-402 (2013).<br>Collagen I (ab34710): validated to react with mouse IHC-P by the manufacturer; applied in mouse IHC-P by Tessier S et al. Dev Biol 455:369-381 (2019).<br>Collagen III (ab7778): validated to react with mouse IHC-P by the manufacturer; applied in mouse IHC-P by Munji RN et al. Nat Neurosci 22:1892-1902 (2019).<br>MMP9 (GTX100458): orthogonal validation by the manufacturer, validated to react and to be applied in mouse IHC-P; applied in mouse IHC-P by Murata K et al. J Nat Med 75 (1):142-155 (2021).<br>Snai1 (13099-1-AP): KD/KO validated by the manufacturer. Validated to react and to be applied in mouse IHC by Wang Y et al., Chemosphere doi: 10.1016/j.chemosphere.2020.128870. Epub 2020 Nov 4.<br>P-cadherin (13773-1-AP): Validated by the manufacturer to react in mouse and in IHC application; also used in Li et al., Int J Biol Sci Mar 10;15(5):953-961. eCollection (2019).<br>E-cadherin (20874-1-AP): KD/KO validated by the manufacturer. Validated to react and to be applied in mouse IHC by Cao N et al., Oncotarget Sep 20;7(38):61093-61106 (2016).<br>Zeb2 (14026-1-AP): KD/KO validated by the manufacturer. Validated to react and to be applied in mouse IHC by Qu Y et al., Eur J Cancer. Nov;49(17):3718-28 (2013).

Eukaryotic cell lines

Policy information about cell lines

Cell line source(s)	293T (ATCC® CRL-3216™)
Authentication	Short tandom repeat (STR) profiling was used to authenticate 293T cell line.
Mycoplasma contamination	293T cell line was tested negative for Mycoplasma.
Commonly misidentified lines (See ICLAC register)	No misidentified cell line was used in this study.

Animals and other organisms

Policy information about studies involving animals; ARRIVE guidelines recommended for reporting animal research

Laboratory animals	C57Bl/6J mouse (4-week-old), K14-Cre-Twist1 mouse (4-week-old), wild type Twist1+/+ mouse (4-week-old), African spiny mouse (Acomys cahirinus, 2-month-old). Both sexes were used.
Wild animals	No wild animal was involved.
Field-collected samples	No field-collected sample was involved.
Ethics oversight	All animal work was performed according to the approved animal protocol, guidelines and regulations for the care and use of laboratory animals of University of Southern California.

Note that full information on the approval of the study protocol must also be provided in the manuscript.
